# Reducing the risk of healthcare‐associated infections through Lean Six Sigma: The case of the medicine areas at the Federico II University Hospital in Naples (Italy)

**DOI:** 10.1111/jep.12844

**Published:** 2017-11-03

**Authors:** Giovanni Improta, Mario Cesarelli, Paolo Montuori, Liberatina Carmela Santillo, Maria Triassi

**Affiliations:** ^1^ Department of Public Health Federico II University of Naples Naples Italy; ^2^ Department of Electrical Engineering and Information Technology Federico II University of Naples Naples Italy; ^3^ Department of Chemical, Materials and Industrial Production Engineering Federico II University of Naples Naples Italy

**Keywords:** healthcare, healthcare services research, healthcare‐associated infections, Lean Six Sigma, public health

## Abstract

**Rationale, aims, and objectives:**

Lean Six Sigma (LSS) has been recognized as an effective management tool for improving healthcare performance. Here, LSS was adopted to reduce the risk of healthcare‐associated infections (HAIs), a critical quality parameter in the healthcare sector.

**Methods:**

Lean Six Sigma was applied to the areas of clinical medicine (including general medicine, pulmonology, oncology, nephrology, cardiology, neurology, gastroenterology, rheumatology, and diabetology), and data regarding HAIs were collected for 28,000 patients hospitalized between January 2011 and December 2016. Following the LSS define, measure, analyse, improve, and control cycle, the factors influencing the risk of HAI were identified by using typical LSS tools (statistical analyses, brainstorming sessions, and cause‐effect diagrams). Finally, corrective measures to prevent HAIs were implemented and monitored for 1 year after implementation.

**Results:**

Lean Six Sigma proved to be a useful tool for identifying variables affecting the risk of HAIs and implementing corrective actions to improve the performance of the care process. A reduction in the number of patients colonized by sentinel bacteria was achieved after the improvement phase.

**Conclusions:**

The implementation of an LSS approach could significantly decrease the percentage of patients with HAIs.

## 1 INTRODUCTION

The health sector has a significant impact on the socioeconomic growth of a nation, and it contributes to public spending, accounting for almost 15% of all government expenditures in the European Union (EU).^1^ It also accounts for 8% of the total European workforce and for 10% of the EU's gross domestic product.^1^ The sector is vital to ensuring the health and wellbeing of the EU population and is at the core of the EU's high level of social protection.^1^ Thus, healthcare companies (and companies that produce related goods and services) must ensure that they perform effectively and efficiently to avoid wasting public money and to protect both patient health and equitable access to services.

Healthcare‐associated infections (HAIs) are recognized worldwide as an important public health problem, and they are of increasing interest to politicians, patients, and the public.^2^

Patients acquire HAIs during treatment; that is, they are neither present nor incubating at the time of admission.^3^ Healthcare‐associated infections also include infections that appear after discharge and occupational infections among healthcare staff.^4^ Healthcare‐associated infections are the most frequent adverse events in healthcare delivery worldwide, and they lead to significant mortality for patients and financial losses for health systems each year.^5^ Thus, the rate of HAIs is an indicator of the healthcare quality provided in hospitals.^5^, ^6^, ^7^

The European Centre for Disease Prevention and Control reports an HAI prevalence of 7.1% and estimates that approximately 4 million patients are affected every year in Europe.^4^ Many countries lack strong surveillance systems for HAIs, which remain a serious problem that no institution or country has solved despite considerable effort.^4^ Annual financial losses due to HAIs are also significant. In Europe, losses include an estimated €7 billion in direct costs alone and an extra 16 million hospital stay days; in the USA, the corresponding cost is $6.5 billion.^5^

Healthcare‐associated infections are therefore widely investigated in healthcare,^8^, ^9^, ^10^ and research has revealed correlations between certain viral infections and bacteria in various pathologies.^11^, ^12^ Several factors cause HAIs, some of which are related to prolonged and inappropriate use of invasive devices and antibiotics, insufficient application of standard and isolation precautions, inadequate environmental hygiene, poor infrastructure, insufficient equipment, lack of standardized procedures, and the absence of local and national guidelines and policies.^5^, ^13^, ^14^, ^15^

Surveillance of HAIs has been recognized as an important component of any comprehensive infection prevention and control program. In 1998, the Italian National Health Plan identified the reduction of HAIs as a priority, and successive studies^16^, ^17^ emphasized the urgent need for a nationwide HAI surveillance plan to provide the Italian National Health System with the tools to prevent and manage HAIs in hospitalized patients.^16^

At present, the monitoring and prevention of HAIs is a priority for the healthcare sector, and reducing the incidence of HAIs is used as an indicator of the quality of service provided. If rigorously implemented, established preventive measures can substantially reduce the number of infections.^18^ Currently, companies' corporate strategies include active and passive systems of epidemiological surveillance to monitor and prevent HAIs.^19^

When these surveillance strategies are integrated with quality improvement principles, techniques, and management tools, infection prevention systems are considerably strengthened and ensure both patient safety and high‐quality patient care.^12^, ^19^, ^20^, ^21^

In fact, healthcare processes can be analysed as business processes, and tools that are used in business contexts can also be adapted for use in healthcare processes. However, the implementation of management tools to healthcare processes is not always easy due to the lack of global, standardized, and repeatable indicators to measure the quality of care.^22^, ^23^, ^24^

Lean Six Sigma (LSS) is a widely implemented management tool and process improvement technique in the healthcare sector.^25^, ^26^ Six Sigma and lean systems have the same goal in that they both seek to eliminate waste and create the most efficient system possible. However, they identify the root cause of waste differently: from a lean perspective, waste comes from unnecessary steps in a process, whereas in the Six Sigma approach, waste results from variation within the process. Lean Six Sigma uses lean methodologies to identify and remove waste, and then uses Six Sigma tools to reduce process variation.^27^, ^28^, ^29^

Thus, LSS integrates both lean and Six Sigma principles and improves the overall performance of a system^30^ by facilitating the identification of causes of deviations from the ideal process, the elimination of these deviations, and, consequently, the enhancement of process performance.

Lean Six Sigma is mainly focused on efficiency outcomes; for instance, it has been utilized to improve operating room efficiency,^31^ reduce patient waiting time in an outpatient department,^32^ improve primary care practices,^33^ and reduce the length of stay associated with liver transplants.^34^

In addition, it has been applied to reduce turnaround time by improving a hospital medical records department,^35^ improve the quality and costs of hip replacement surgery,^36^ increase patient satisfaction,^37^, ^38^ and reduce hospital registration processing times.^39^, ^40^ It has also been applied in emergency departments in various ways, such as to reduce hemolysis,^41^ decrease departmental inefficiencies and their costs,^42^ reduce waiting times,^43^ and improve patient satisfaction.^44^ Furthermore, it has been successfully used to reduce the incidence of catheter‐related bloodstream infections in an intensive care unit^26^ and to reduce surgical site infections.^45^

In this approach, healthcare professionals (physicians, technicians, physician assistants, nurses, clinical officers, and operating department practitioners), regardless of their area of expertise, are expected to be able to analyse and solve problems efficiently and effectively.^46^, ^47^ That is, they are expected to have both technical and managerial competences.^46^, ^47^

We recently applied LSS to reducing the number of patients affected by sentinel bacterial in surgery departments,^48^ which resulted in a significant reduction in both the number of hospitalization days and the number of patients with HAIs.

The implementation of this intervention in the general surgery departments resulted in a significant reduction in both the number of hospitalization days and the number of patients affected by Hn and significant reduction in both the number of hospitalization days and the number of patients affected by HAI.

The aim of the present study, which is part of the same *LSS Methodology to Reduce Healthcare Infections* project, is to apply LSS to clinical medicine areas (general medicine, pulmonology, oncology, nephrology, cardiology, neurology, gastroenterology, rheumatology, and diabetology) to enable the identification of variables that influence HAI risk in these areas and to compare them with HAI risk in surgery departments. To exploit the wide variety of LSS tools available and make the study robust, we applied different tools from those considered in our previous study.^48^

This analysis was conducted at Federico II University Hospital in Naples (Italy) from January 2011 to December 2016 on 28,000 patients. We were able to develop corrective actions to improve the overall performance of the services examined.

In accordance with the literature and with national and regional legislation, the University Hospital in Naples (Italy) has adopted an integrated strategy to monitor and prevent the occurrence of infections that can cause diseases. The integrated application of this monitoring strategy along with the LSS methodology allows for improved performance of the care process by reducing the incidence of infections and therefore decreasing the risk of HAIs.

The purpose of this study is to reduce the risk of HAIs in various areas of clinical medicine by using LSS tools to improve healthcare processes.

## 2 METHODS

Consistent with the guidelines of the Helsinki Declaration of 1975 (revised in 2000) concerning experiments involving human participants, this study met the criteria for operational improvement activities and was approved by the University's Research Committee.

In accordance with the LSS methodology, the data analysis is structured according to the define, measure, analyse, improve, and control cycle.

- Define: Identifying the study

The define phase started with a clear definition of the LSS project aim, i.e., to reduce the risk of HAIs in clinical medicine areas, and the team responsible for its implementation. The team leader was the director of the Public Health Department; furthermore, several physicians and engineers were involved in the project.

The LSS methodology was applied to clinical medicine areas (general medicine, pulmonology, oncology, nephrology, cardiology, neurology, gastroenterology, rheumatology, and diabetology), and data on 28,000 hospitalized patients were collected between January 2011 and December 2016. Data regarding hospitalization days, infections, and number of diagnostic and therapeutic procedures were collected for each patient by using the departmental information system. Among infected patients, the most prevalent sentinel bacteria were determined. The preintervention (January 2011 to December 2014) and postintervention (January 2015 to December 2016) phases were compared to analyse effects of the project.

In accordance with the Six Sigma approach, the critical‐to‐quality characteristic (i.e., the dependent variable of the process analysed) was identified by the team members as the number of patients with positive test results for HAI (specifically, the number of patients for which at least 1 positive biological sample of sentinel bacterium was reported by the microbiology unit).

- Measure: Data collection

The study data were extracted from the hospital database, which records information about patients' hospital discharge and infection monitoring (number of infections and type of sentinel bacteria). These data provide information concerning the independent variables of the process under investigation, i.e., patients' personal data (age and gender), number of treatments per patient, patient hospitalization duration (days), and number of days before patient admission. To characterize the data sample for the study, statistical descriptive analyses were conducted by using Fisher's and chi‐square tests.

- Analyse: Analysis of causes

The Analyse stage was carried out by using tools such as brainstorming and cause‐effect diagrams. First, correlations between the dependent variable (colonization) and the independent variables (patients' personal data (age and gender), number of treatments per patient, patient hospitalization duration (days), and number of days before patient admission) were evaluated. The objective of this stage was to find the root causes of risks so that they could be eliminated to improve the process. In this stage, the team used a simple cause‐effect diagram. The diagram was invented in the 1960s by Ishikawa^49^ and is still applied today in problem‐solving processes. During the brainstorm process, the team discussed the potential reasons for rejecting or considering causes based on substance and reasonability. Then, expert opinion was obtained by administering a questionnaire^48^ to members of the Hospital Infection Committee to investigate the protocols, procedures, and precautionary actions adopted by the healthcare staff to limit the risk of HAI. The questionnaire highlighted a lack of standardized procedures to prevent infections, as well as a lack of information about HAIs, and allowed for the identification of corrective measures to improve the process.

- Improve: Improvement.

Brainstorming aimed to thoroughly discuss the causes and problems that came to light in the *Analyse phase*, allowing the project team to identify and implement corrective measures (selection and monitoring of clinical pathways, more appropriate adoption of clinical procedures, and early identification of the colonized patients) aimed at overcoming the revealed problems.

- Control: Implementation of the control and feedback system

The corrective actions can be evaluated to determine whether they lead to performance improvements in the analysed process.

The efficacy and efficiency of the implemented improvement measures (adoption of clinical procedures, healthcare staff formation, information about HAIs, and monitoring clinical pathways) were measured over a 2‐year period to investigate the effectiveness of the interventions over the long term. Given the physiological processes associated with HAIs, improvement was measured in the reduction of the number of patients colonized by sentinel bacteria and therefore at risk of contracting HAIs. To continuously improve the process and maintain a high standard of quality, a quality control plan was implemented. The plan was divided into the following phases:

1. Process/procedure standardization: implementing standard precautions, e.g., introducing best practices for hand hygiene, can prevent HAIs.
2. Regular monitoring: the status and number of patients colonized by sentinel bacteria was monitored.
3. Evaluation of corrective actions: evaluation involved data analysis, brainstorming activities, identification, and control of key performance indicators (e.g., number of colonized patients, number of standardized procedures, and length of hospital stay).
4. Continuous improvement: the staff training system and the management of patient data were improved through staff education and accountability, which are essential to making healthcare providers and patients aware of risks and consequences of HAIs and to promoting strategies to prevent them.
5. Collection of data: the collection and preservation of data related to each infection are useful for adapting and validating the implemented surveillance protocols, conducting research and prevalence surveys, and identifying solutions to improve the healthcare services related to a particular infection.
6. The management of quality controls: ensuring high quality and performance involves adopting proper quality control systems and procedures during each phase of the process.

## 3 RESULTS

This section details the implementation of the phases described in the previous section.

- Define

**Figure 1:**
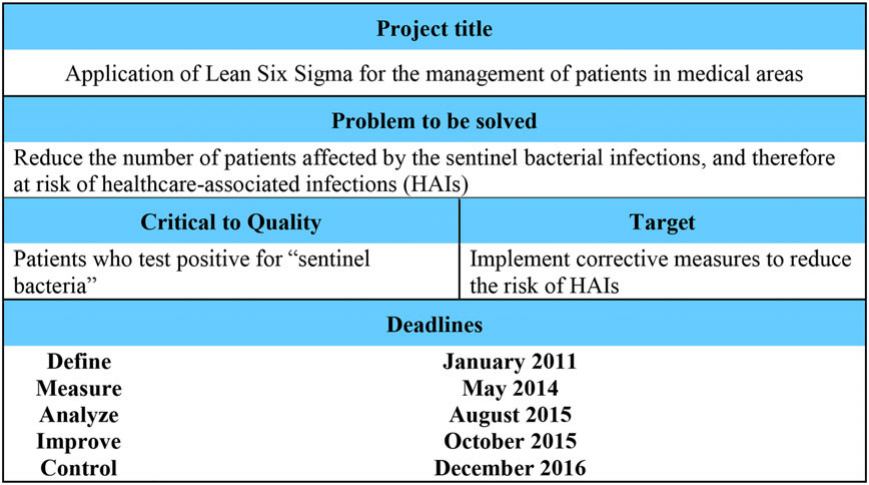
Lean Six Sigma project statement

This phase was characterized by the development of the project statement (Figure 1), which clearly defined the analysed process and enabled the identification of risk factors and therefore the critical‐to‐quality characteristic. After discussion within the project team and a literature survey, the goal of this project was defined as *the reduction of sentinel bacteria colonization*. This statement also helped define the Gantt activities for each phase of the LSS approach (define, measure, analyse, improve, and control). Specifically, the team members defined the goal of the project as the *reduction of the number of patients affected by sentinel bacterial and therefore at risk of HAIs*.

- Measure

**Figure 2:**
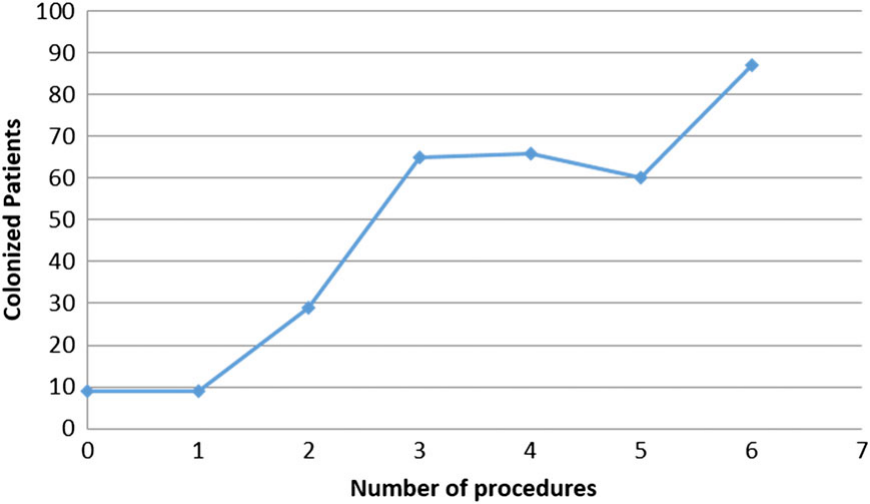
Scatter plot of colonized patients versus number of procedures

Figure 2 highlights the correlation between the number of colonized patients and the number of treatments that those patients received, which are here generically called “procedures,” i.e., the number of diagnostic and/or therapeutic procedures administered to each patients within the observation period. The estimated percentage of colonized patients was 0.36% (325 colonized patients), which was similar to the share (0.37%) observed in surgery departments.^48^ Chi‐square tests revealed a correlation between the number of procedures and the risk of HAIs. Fischer's tests did not provide evidence of a correlation between HAI and the number of hospitalization days.

- Analyse

**Figure 3:**
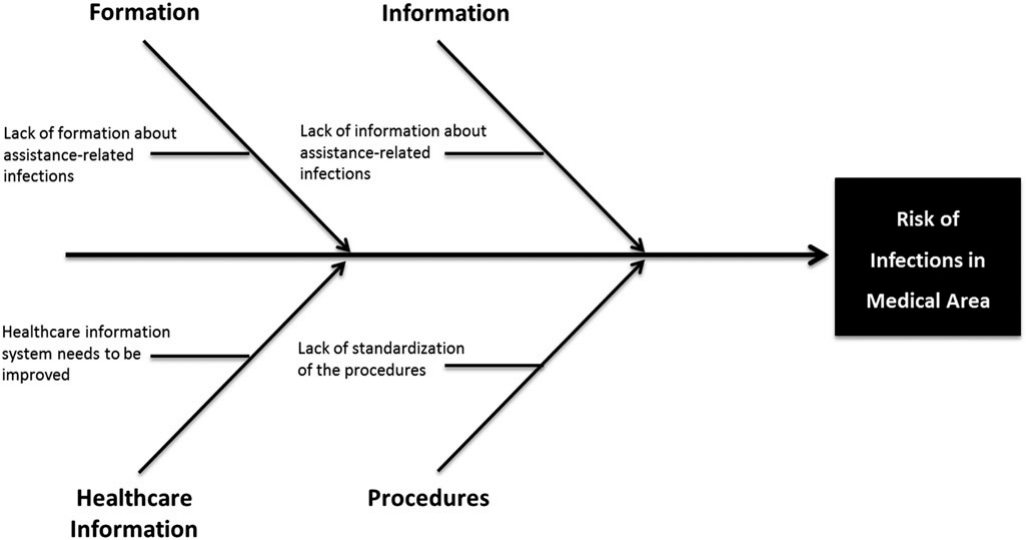
Cause‐effect diagram

During this phase, the data collected and measured in the previous phase were analysed. The distribution of sentinel bacteria was evaluated to determine the incidence of each bacterium in the sample. Figure 2 confirms a strong positive correlation between the number of patients colonized and the number of procedures, as previously highlighted for surgery departments. Therefore, a cause‐effect diagram (Figure 3) was developed to identify the causes of infections and possible actions for process improvement. The causes were categorized into 4 different areas: (1) information for healthcare staff about procedures to reduce HAIs, (2) information about factors determining the risk of HAIs, (3) healthcare information systems to monitor and collect data on hospital infections, and (4) availability of standardized procedures to reduce the risk of HAIs. Because the *Measure phase* revealed no correlations between HAIs and demographic data, nor with hospitalization days, these factors were not included in the cause‐effect diagram.

- Improve

The previous phases and the questionnaire results allow for the identification of causes and the implementation of corrective actions to optimize the examined process.

The following table (Table 1) summarizes the corrective actions identified and implemented in this process to optimize performance and reduce the risk of HAI.

- Control

**Table 1:** Causes influencing the risk of infections and possible solutions

Causes	Solution
Lack of standardization of procedures	Application of evidence‐based medicine to select clinical pathways for patients
Lack of standardization of procedures	More appropriate adoption of clinical procedures
Healthcare information system that could be improved	More accurate and careful collection of data related to patients' clinical pathways
Lack of training and information with respect to health related infections	Early identification of colonized patients

**Figure 4:**
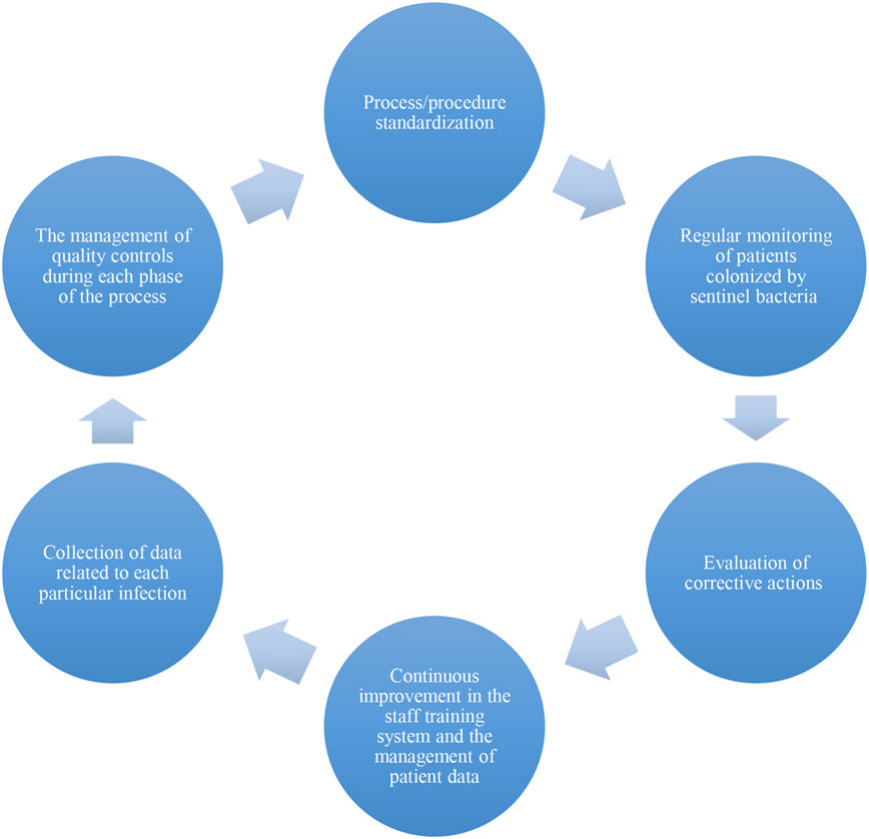
Quality control plan cycle

To continuously improve the process and maintain a high standard of quality, a quality control plan was implemented; this plan included the phases shown in Figure 4.

As a result of these improvements, both the number of colonized patients and the corresponding duration of hospitalization have been significantly reduced. In particular, the percentage of colonized patients was reduced from 0.36% to 0.19% (only 25 patients of analysed patients).

## 4 DISCUSSION AND CONCLUSIONS

To improve hospital safety and performance, it is possible to adapt the tools and methods of business management, such as the LSS approach, to the healthcare sector. This study demonstrates that this approach allows healthcare professionals to increase process efficiency, reduce healthcare costs, and improve the quality of service and therefore of the healthcare system.

Having been applied to surgery departments, LSS was examined in clinical medicine areas (as mentioned in section 2), with the following aims: recognizing the main factors leading to sentinel bacteria colonization, therefore increasing the risk of HAI, and identifying and implementing corrective actions to reduce the risk of HAIs in hospitalized patients and to improve the performance of the entire care process.

Thus, this study presents an application of management tools to the healthcare sector. Specifically, these tools were applied in the areas of clinical medicine (as mentioned in section 2).

After the implementation of corrective measures, the percentage of colonized patients was reduced from 0.36 to 0.19%, confirming an efficacy of LSS comparable with that obtained for surgery departments. For completeness, a comparison of the 2 fundamental studies of the Lean Six Sigma Methodology to Reduce Healthcare Infections project is provided in Table 2.

**Table 2:** Comparison between the 2 fundamental studies of the project

Lean Six Sigma Methodology to Reduce Healthcare Infections Project Federico II University Hospital in Naples		
Comparison of the 2 fundamental Studies of the Project		
	First Study	Second Study
Area of application	Surgery departments	Medicine areas
Number of analysed patients	20,000	28,000
Analysed period	January 2011 to December 2014	January 2011 to December 2016
Define phase	Statistical tools: project charter Gantt diagram SIPOC analysis critical‐to‐quality (CTQ) definitions	Statistical tools: project charter Gantt diagram CTQ definitions
Measure phase	Patient data are extracted from QUANI, a program developed by Bim Italia to record patients' hospital discharge data and flow data for the monitoring of sentinel bacteria. The used statistical tools are scatter box plot	Data for the study were extracted from the hospital database, which is able to record patients' hospital discharge data as well as flow data for the monitoring of sentinel bacteria. The used *statistical tools* are histograms, chi‐square tests, and Fisher tests.
Analyse phase	The used statistical tools are control chart histograms, chi‐square tests, and Fisher tests. Additionally, an Ishikawa fishbone diagram was developed to determine the root causes for the identified problem.	Analysis of the data collected during the measure phase. The used statistical tool is cause‐effect diagram and brainstorming sessions to deepen and validate the analysis of the root causes with the support of expert and healthcare staff.
Improve phase	Expert advice was obtained by administering a questionnaire to members of the Hospital Infection Committee that would allow them to indicate any necessary corrective measures to improve the process. A table summarizes all of the causes validated through the questionnaire and the corresponding corrective actions to be implemented in the process to optimize the process performance and reduce the risk of HAIs.	Expert advice was obtained by administering the same questionnaire. The previous phases and the questionnaire results allow for the identification of causes and the implementation of corrective actions to optimize the examined process.
Control phase	To control the course of the process, monitoring was performed by using process indicators.	To continuously improve the process and maintain a high standard of quality, a quality control plan was implemented.
Percentage of colonized patients	0.37%	0.36%
Implementing corrective actions	The application of corrective actions leads to a reduction in the percentage of colonized patients from 0.37% to 0.21%. Furthermore, the corrective actions significantly reduce the mean (SD) number of days of hospitalization from 45 (30.78) (with a data distribution approximately 2*σ*) to 36 (5.68) (with a data distribution approximately 3*σ*)	The percentage of colonized patients was reduced from 0.36% to 0.19% (only 25 patients of total patients analysed).

The proper allocation of resources, including waste reduction, is essential in the healthcare sector. The lack of resources to satisfy healthcare needs, together with the need for excellent performance and safe healthcare, are reasons for finding and adopting managerial strategies to minimize costs and reduce waste while improving the quality of services provided. Management tools must be adopted to ensure proper analysis of complex hospital systems and to improve and monitor these from both clinical and economic perspectives. The proposed strategy and quality control cycle could be implemented to continuously improve healthcare processes and ensure high quality standards.

This study could be improved by extending the statistical analysis and using other LSS tools, such as Pareto charts and other tests to evaluate the correlations between HAIs and parameters such as the type of intervention, comorbidities, allergies, and other factors that could also affect the risk of HAIs. However, these limitations could be usefully addressed in future studies applying LSS to clinical practice. A multicenter study involving 2 or more hospitals could be of great interest to test LSS efficacy in different environments and to assess the validity of corrective measures and standardized procedures to improve 1 or more healthcare process.

## CONFLICT OF INTEREST

The authors declare no conflict of interest.

## APPENDIX 1

### Research checklist^i^

**Table:**

Title and Abstract	
Title	Reducing the risk of healthcare‐associated infections through Lean Six Sigma: the case of the medicine areas at the Federico II University Hospital in Naples (Italy)
Abstract	Rationale, aims, and objectives: The use of a Lean Six Sigma (LSS) has been recognized as an effective management tool to improve healthcare performance. Here, LSS is adopted to reduce the risk of healthcare‐associated infections (HAIs), a critical quality parameter in the healthcare sector. Methods: LSS was applied to the area of clinical medicine (including general medicine, pulmonology, oncology, nephrology, cardiology, neurology, gastroenterology, rheumatology, and diabetology), and data regarding HAIs were collected on 28,000 hospitalized patients between January 2011 and December 2016. Following the LSS DMAIC (define, measure, analyse, improve, and control) cycle, factors influencing the risk of HAIs were identified by using typical LSS tools (statistical analyses, brainstorming sessions, and cause‐effect diagrams). Finally, corrective measures to prevent HAIs were implemented and monitored over a year after implementation. Results: LSS proved to be a useful tool to identify variables affecting the risk of HAIs and to implement corrective actions to improve the performance of the care process. Reduction in the number of patients colonized by the sentinel bacteria was achieved after the improvement phase. Conclusions: The LSS approach produced a significant decrease in the percentage of infected patients in hospitals.
Introduction	
Problem description	Currently, the monitoring and prevention of HAIs represents a priority for the healthcare sector, and reducing their incidence is a quality indicator of the services provided.
Available knowledge	Process improvement can be achieved through by developing collaborative applications and adoption of ontological relations. Among the most widespread solutions to minimize cost and improve service quality, LSS seems to be one of the most innovative and effective approaches in “operational excellence.”
Specific aims	This work aims to apply the LSS methodology with different statistical analyses to enable the identification of variables that influence the risk of HAI at Federico II University Hospital in Naples (Italy) in medicine areas and thereby permit the implementation of corrective actions to improve the overall performance of the services provided.
Methods	
Context	The project was developed at the Federico II University Hospital in Naples (Italy). Consistent with a typical Lean Six Sigma improvement process, the DMAIC method has been adopted to perform the study.
Intervention(s)	The research was conducted by a multidisciplinary team and according to the DMAIC cycle after an in‐depth understanding of the problem achieved through process mapping, data measures, and brainstorming activities, to optimize the main procedures of the care process, reducing wastes and delays.
Study of the intervention(s)	The causes of infection occurrences were analysed by using LSS tools. Finally, expert advice was obtained by administering a basic questionnaire to members of the Hospital Infection Committee that allowed the identification of any corrective measures needed to improve the investigated process.
Measures	Data for the study were extrapolated from the hospital database, which is able to record patients' hospital discharge data as well as flow data for the monitoring of sentinel bacteria. These data provide information concerning the independent variables of the process under investigation, i.e., patients' personal data (age and gender), the numbers of treatments for patients, patient hospitalization durations (days), and the number of days before patient admission.
Results	
Results	As a result of these improvements, both the number of colonized patients and the corresponding duration of hospitalization have been significantly reduced. In particular, the percentage of colonized patients was reduced from 0.36% to 0.19% (only 25 patients of the total analysed patients). We also tested a decrease in the mean (SD) number of days of hospitalization, which amounted to 25 with a data distribution approximately 3*σ*.
Discussion	
Summary	Already applied to the surgery departments, the LSS methodology is used to confirm the ability also in medicine areas, with the aim of recognizing the main factors leading to sentinel bacteria colonization and therefore increasing the risk of HAI and identifying and implementing corrective actions to reduce the risk of HAI in hospitalized patients and improve the performance of the entire care process.
Conclusions	After the implementation of the corrective measures, the percentage of colonized patients was reduced from 0.37 to 0.19%, confirming that the efficacy of LSS in medicine is comparable with that in the surgery department study. In particular, in this study, the longer observation period and the higher number of analysed patients have confirmed and optimized the statistical analysis.
